# Construction of Two Alternative Polyadenylation Signatures to Predict the Prognosis of Sarcoma Patients

**DOI:** 10.3389/fcell.2021.595331

**Published:** 2021-06-14

**Authors:** Chuan Hu, Chuan Liu, Jianyi Li, Tengbo Yu, Jun Dong, Bo Chen, Yukun Du, Xiaojie Tang, Yongming Xi

**Affiliations:** ^1^Department of Orthopaedic Surgery, The Affiliated Hospital of Qingdao University, Qingdao, China; ^2^Graduate School, China Medical University, Shenyang, China; ^3^Department of Sports Medicine, The Affiliated Hospital of Qingdao University, Qingdao, China; ^4^Department of Orthopedics, Shandong Provincial Hospital Affiliated to Shandong First Medical University, Jinan, China; ^5^The First Clinical College, Wenzhou Medical University, Wenzhou, China

**Keywords:** alternative polyadenylation, sarcoma, overall survival, progress free-survival, nomogram

## Abstract

**Background:**

Increasing evidence indicates that alternative polyadenylation (APA) is associated with the prognosis of cancers.

**Methods:**

We obtained gene expression and APA profiles of 259 sarcoma patients from the TCGA dataportal and TC3A database, respectively. The prognostic signatures, clinical nomograms, and regulatory networks were studied by integrated bioinformatics analyses. Then, the immune cell infiltration profile was obtained from the ImmuCellAI. The association between APA-based signature and immune cells was studied.

**Results:**

A total of 61 and 38 APA events were identified as overall survival (OS)- and progress free-survival (PFS)-related biomarkers, respectively. Two signatures were generated. The area under the curves (AUC) values of OS signature were 0.900, 0.928, and 0.963 over 2-, 4-, and 6-years, respectively. And the AUC values of PFS signature at 2-, 4-, and 6-years were 0.826, 0.840, and 0.847, respectively. Overall and subgroup analyses indicated that high-risk patients had a worse prognosis than low-risk patients (all *p*-values < 0.05). In addition, immunomics analyses indicated that there are different patterns of immune cell infiltration between low- and high-risk patients. Furthermore, two clinical-APA nomograms were established and the C-indexes were 0.813 and 0.809 for OS nomogram and PFS nomogram, respectively. Finally, two APA regulatory networks were constructed. FIP1L1-VPS26B was identified as a key regulating relationship and validated in the pan-cancer analyses.

**Conclusion:**

In this study, we identified prognostic predictors based on APA events with high accuracy for risk stratification in sarcoma patients and uncovered interesting regulatory networks in sarcoma that could be underlying mechanisms. This study not only provides novel potential prognostic biomarkers but promote precision medicine and provide potential novel research interests for immunotherapy.

## Introduction

Sarcomas are a heterogeneous group of mesenchymal malignancies that can develop at any age, comprising approximately 1% of all adult malignancies and 15% of pediatric malignancies (Von Mehren et al., 2018). Although the incidence of sarcoma is relatively rare, more than 10,000 patients are diagnosed with soft tissue sarcomas in the United States and 40,000 in China each year (Yang et al., 2019). Surgery, radiotherapy, and chemotherapy are three mainstream treatments for sarcoma patients that have shown progressive effects (Nussbaum et al., 2016; Albertsmeier et al., 2018; Thanindratarn et al., 2019). Unfortunately, the prognosis of sarcoma patients is still unsatisfactory due to local recurrence and distant metastases (Cipriano et al., 2020). Therefore, it is urgent to develop a reliable prognostic predictor for guiding clinical practice. Based on either clinical data, gene expression profile, or tumor-infiltrating immune cell, several prognostic models have been developed for sarcoma patients before (Callegaro et al., 2019; Huang et al., 2019; Gu et al., 2020). Nevertheless, there have not been any reliable models due to the complexity and heterogeneity of sarcoma.

Alternative polyadenylation (APA) is an important post-transcriptional regulation mechanism, which occurs in >70% of human genes (Mayr and Bartel, 2009; Hoque et al., 2013). It was found that APA plays an essential role in protein diversification, mRNA stability, mRNA nuclear export and repression of gene expression by producing mRNAs with different 3′ untranslated regions (3′ UTRs) and/or encoding variable protein isoforms (Edwalds-Gilbert et al., 1997; Tian and Manley, 2017). Therefore, from the perspective of epigenetics, once APA is dysregulated, it will cause diverse pathological processes, such as cancer, viral infection, amyotrophic lateral sclerosis, and so on (Chen et al., 2017).

Nowadays, the deregulation of APA has caused widespread interest in cancer research, because APA generates mRNA 3’ UTR isoforms with potentially different stabilities, subcellular localizations, translation efficiencies, and functions. In recent years, with the rapid development of high-throughput sequencing technology, genome-wide profiling for APA events has become a reality. In 2018, Li et al., (Xiang et al., 2018) have completed an analysis of pan-cancer analysis that helps us to understand the regulatory mechanisms and functional consequences of APA alterations in tumorigenesis. In total, 17 tumor types were studied and a series of important roles of APA in the tumor were discovered, such as gene expression regulation and cellular pathway remodeling (Xiang et al., 2018). Recently, Venkat et al. (2020) firstly performed a cancer-specific analysis and found that APA was an independent prognostic biomarker for pancreatic ductal adenocarcinoma patients. Generally, as with other well-researched post-transcriptional regulation mechanisms like alternative splicing and mRNA m6A methylation, APA also plays a vital role in the genesis, progress, and prognosis of cancers. Despite the effect of APA was preliminarily confirmed in this research, there were few attempts to study the role of APA for sarcoma, and no APA-based signature was constructed for such patients.

In the present study, a comprehensive analysis of APA events was performed based on a large cohort from the TCGA-SARC dataset. The prognostic value of APA events for sarcoma patients was uncovered, and two APA-based signatures were constructed. We further explored the potential relationship between APA signatures and clinicopathological data and developed two clinical-APA nomograms. Finally, we established the regulatory network between APA events and APA factors to elucidate the underlying mechanisms.

## Materials and Methods

### Data Acquisition

The gene expression files and clinical data of TCGA-SARC were obtained from the UCSC Xena browser^1^. Additionally, the Percentage of Distal polyA site Usage Index (PDUI) value was used to quantify each APA event, and the data of PDUI of TCGA-SARC was downloaded from the TC3A database (Feng et al., 2018). According to the definition in published research, the PDUI score quantifies the relative poly (A) site usage for that gene in the sample by computing the abundances of 3’-UTR long and short forms (Venkat et al., 2020). Genes favoring distal PAS usage (long 3’ UTRs) have PDUI scores near 1, whereas genes favoring proximal PAS usage (short 3’ UTRs) have PDUI scores near 0 (Venkat et al., 2020). To construct as credible a set of APA events as possible, only APA events that met the following criteria were included in this study: (Von Mehren et al., 2018) percentage of samples with PDUI value ≥75%; (Yang et al., 2019) average PDUI value ≥0.05 (Li et al., 2019; Zhang et al., 2020b). All data were downloaded from the public databases hence it was not required to obtain additional ethical approval for our study.

### Survival Analysis and Enrichment Analysis

To comprehensively understand the role of APA event in sarcoma, two primary endpoints were studied, including overall survival (OS) and progress-free survival (PFS). According to the PDUI value, all patients were stratified into low- and high-PDUI groups by the median cut. Then, the univariate Cox analysis was performed to identify prognostic APA events for sarcoma patients, including OS- and PFS-related APA events. To obtain the robust prognostic APA events, only APA events with a *p*-value < 0.01 in the univariate Cox analysis were selected for further analyses (Zhu et al., 2018). To further understand the mechanisms involved in APA events affecting the prognosis of patients, the parent genes of identified APA events were then incorporated into Gene Ontology (GO) enrichment analysis. The enrichment analyses were performed in the Metascape^2^.

### Establishment and Evaluation of APA-Based Signatures

To avoid overfitting among prognostic APA events, a machine learning algorism called Least absolute shrinkage and selection operator (LASSO) was used to filter significant APA events. Finally, the multivariate Cox analysis was performed to identify independent prognostic APA events. A robust prognostic signature is valuable for prognostic prediction, clinical management, and accurate clinical trial. Previous research indicated that APA events can serve as effective prognostic biomarkers for survival prediction and that the power of APA events exceeded clinical covariates (Venkat et al., 2020; Zhang et al., 2020b). Therefore, based on corresponding independent prognostic APA events, two prognostic signatures were established, including OS and PFS signatures. To show the discrimination of signatures, a time-dependent receiver operating characteristic (ROC) curve with area under the curve (AUC) was generated (Heagerty et al., 2000). In addition, according to the median of risk score, all patients were stratified into low- and high-risk groups, and the survival curve with a log-rank test was used to show the distinct prognosis between low- and high-risk groups.

To confirm the stability of APA-based signatures in different subgroups, subgroup analyses were performed. Kaplan-Meier survival curves of low- and high-risk patients in several clinical subgroups, including age, sex, histological type, metastatic status, tumor site, surgical margin status, and multifocal status, were constructed. A log-rank test was used to compare the prognostic difference between patients in low- and high-risk groups.

### GSVA Analysis and Immune Cell Infiltration

Gene set variation analysis (GSVA) is a non-parametric and unsupervised method that is commonly used to estimate the variation of the pathway and biological process activity in expression cohort samples (Hänzelmann et al., 2013). The gene set of “c2.cp.kegg.v7.1.symbols” was downloaded from MSigDB database for running GSVA analysis. Adjusted *P* with value less than 0.05 was considered as statistically significance. Previous studies reported that post-transcriptional regulation mechanism plays an important role in the formation of the tumor microenvironment (Li et al., 2019; Yi et al., 2020; Zhang et al., 2020a). Therefore, we further elucidated the association between immune cell infiltration and APA signatures. The immune cell infiltration profile was obtained from the ImmuCellAI (Miao et al., 2020). The difference between low- and high-risk groups were evaluated by Wilcoxon tests.

### Development of Clinical-APA Nomogram for Sarcoma Patients

Nomogram is one of the effective tools for clinical practice, which was widely used as a prognostic device for cancer patients (Iasonos et al., 2008). Therefore, to confirm that APA-based signatures are an independent prognostic predictor for sarcoma and to develop two clinical-APA nomograms, the clinical data, including age, sex, race, tumor site, histological type, distant metastatic status, postoperative radiotherapy, pharmacotherapy, multifocal status, and surgical margin resection status were extracted for further analyses. The univariate Cox analysis was used to identify prognostic variables, and variables with a *p* < 0.05 were incorporated into the multivariate Cox analysis. Then, two nomograms were developed by incorporating several independent predictors. A concordance index (C-index) was used to show the discrimination of nomograms, and the calibration curve was selected to show the calibration of nomograms (Iasonos et al., 2008).

### Correlation Network Between APA and APA Factors

The 3′ end-processing machinery is composed of multiple protein factors and several APA factors were confirmed as vital regulatory factors for APA. Based on a published articles, 28 core APA factors were included (Tang et al., 2016; Qiu et al., 2017; Xiang et al., 2018; Chatrikhi et al., 2019; Jia et al., 2019; Zhang et al., 2019b). The expression of 28 APA factors was downloaded from the UCSC Xena browser (see text footnote 1). The prognostic role of all APA factors was investigated, and OS- and PFS-related APA factors were used for further correlation analyses. Pearson correlation analysis was used to determine the correlation coefficient between the PDUI of APA events and the expression of APA factors and to identify the potential regulatory networks between them (| r| > 0.2 and *p* < 0.05). The regulatory networks was visualized by Cytoscape 3.7.2 (Shannon et al., 2003).

### Statistical Analyses

In the present study, all statistical analyses were performed with R software (version 3.6.1). Except for the special instructions, a *p*-value < 0.05 (two sides) was considered as statistically significant. Univariate, LASSO, and multivariate Cox analyses were used to select independent prognostic APA events by “survival” and “glmnet” packages. The “survivalROC” package was used to develop time-dependent ROC curves, and corresponding AUC values were generated simultaneously. The survival curve was generated by a “survminer” package. In addition, Pearson correlation analysis was used to identify the potential regulatory network between APA events and APA factors (| r| > 0.2 and *p* < 0.05).

## Results

### Overview of APA Events Profiling in Sarcoma

According to the aforementioned criterion, a total of 259 primary sarcoma patients were included in our study. The mean age was 60.71 ± 14.59, and the average follow up time was 3.26 years (range: 0.04–15.56 years). In total, 98 patients died during the follow-up duration and 153 patients progressed. For APA events, 8864 APA events were detected for the TCGA-SARC cohort in TC3A database. A total of 2179 APA events were excluded because more than 25% of patients lacked PDUI of these APA events or the average of PDUI value < 0.05. Finally, 6685 APA events were used for further analyses.

### Identification of Prognosis-Related APA Events and Enrichment Analysis

**FIGURE 1:**
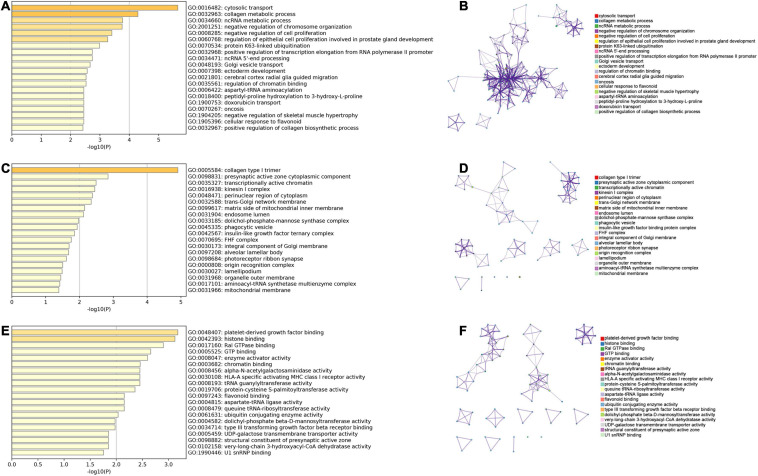
GO analysis of parent genes for prognostic APA events. **(A,B)** The result of biological process of in the GO analysis. **(C,D)** The result of cellular component of in the GO analysis. **(E,F)** The result of molecular function of in the GO analysis. GO, Gene Ontology; APA, alternative polyadenylation.

According to the median of PDUI, all patients were stratified as low- and high-groups for each APA event. The univariate Cox analysis indicated that 61 and 38 APA events were OS- and PFS-related biomarkers, respectively (*p* < 0.01) (Supplementary Tables 1, 2). The enrichment of the GO analysis is illustrated in Figure 1, which showed that specific GO categories were significantly related to sarcomas, like cytosolic transport, collagen metabolic process and negative regulation of chromosome organization.

**FIGURE 2:**
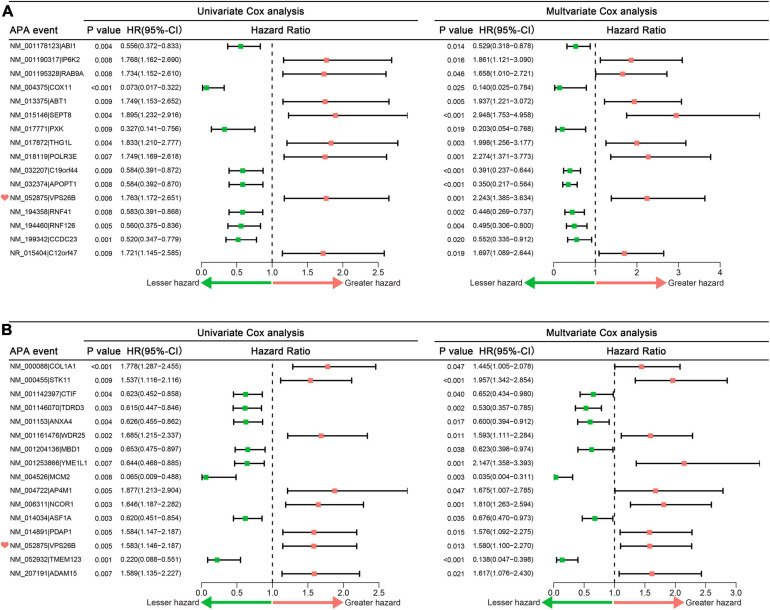
Forrest plots of hazard ratios of prognosis associated APA events in sarcoma patients. **(A)** Univariate and multivariate Cox analysis results of independent OS-related alternative polyadenylation events. **(B)** Univariate and multivariate Cox analysis results of independent PFS-related alternative polyadenylation events. APA, alternative polyadenylation; OS, overall survival; PFS, progress free-survival.

Furthermore, LASSO analysis excluded 22 OS-related APA events and 7 PFS-related APA events (Supplementary Figures 1A–D). Finally, 16 and 16 APA events were confirmed as independent OS- and PFS-related biomarkers, respectively (Figures 2A,B). Interestingly, we found that the APA event of VPS26B was the overlapping independent prognostic APA event between OS and PFS.

### Construction of APA-Based Signatures

**FIGURE 3:**
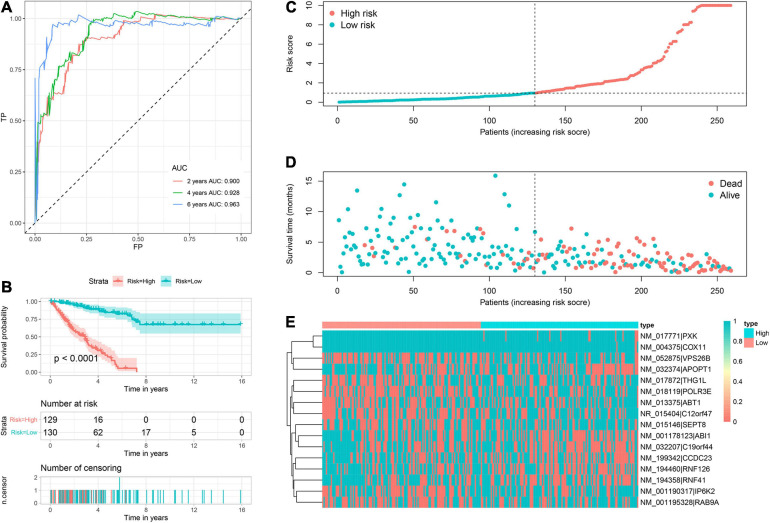
Prognostic signature to predict OS of sarcoma patients. **(A)** ROC curves of OS signature. **(B)** Survival curve showed that high-risk patients were worse OS than low-risk patients. **(C)** Risk score distribution of 259 sarcoma patients. **(D)** OS status of 259 sarcoma patients. **(E)** Heatmap showed the distribution of PDUI of 16 OS-related APA events in low- and high-risk patients. OS, overall survival; ROC, receiver operating characteristic; APA, alternative polyadenylation.

**FIGURE 4:**
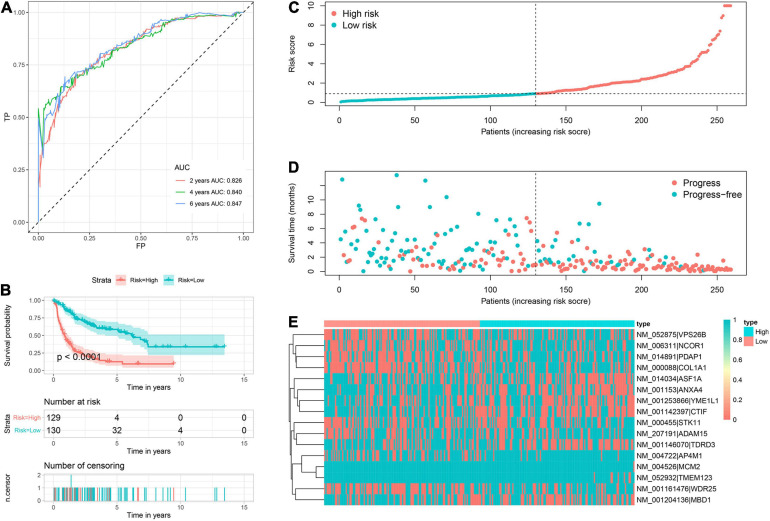
Prognostic signature to predict PFS of sarcoma patients. **(A)** ROC curves of PFS signature. **(B)** Survival curve showed that high-risk patients were worse PFS than low-risk patients. **(C)** Risk score distribution of 259 sarcoma patients. **(D)** PFS status of 259 sarcoma patients. **(E)** Heatmap showed the distribution of PDUI of 16 PFS-related events in low- and high-risk patients. PFS, progress free-survival; ROC, receiver operating characteristic; APA, alternative polyadenylation.

Based on corresponding independent prognostic APA events, two prognostic signatures were constructed to predict the OS and PFS, respectively. The AUC values of OS signature for 2-, 4-, and 6-years were 0.900, 0.928, and 0.963, respectively (Figure 3A). All AUC values were up to 0.900, indicating the great discrimination of this signature. In addition, the AUC values of PFS signature for 2-, 4-, and 6-years were 0.826, 0.840, and 0.847, respectively, which also suggested favorable discrimination (Figure 4A). According to the median of the risk score, all patients were stratified into low- and high-risk groups. Log-rank tests showed that the patients in the high-risk group has worse OS and PFS than the low-risk patients (Figures 3B, 4B). The distribution of risk score (Figures 3C, 4C), prognostic status (Figures 3D, 4D), and PDUI of each sample (Figures 3E, 4E) were visualized to facilitate the understanding of prognostic signatures. Furthermore, we compared the distribution of clinical covariates across high- and low- risk groups (Supplementary Figure 2). For the OS signature, the distribution of multifocal indicator, margin status, and metastasis were significantly different between two risk groups (Supplementary Figure 2A). Additionally, for the PFS signature, the distributions of age, margin status, and metastasis were significantly different between two risk groups (Supplementary Figure 2B).

### The Differences of GSVA Analysis and Immune Cells Between High- and Low-Risk Groups

**FIGURE 5:**
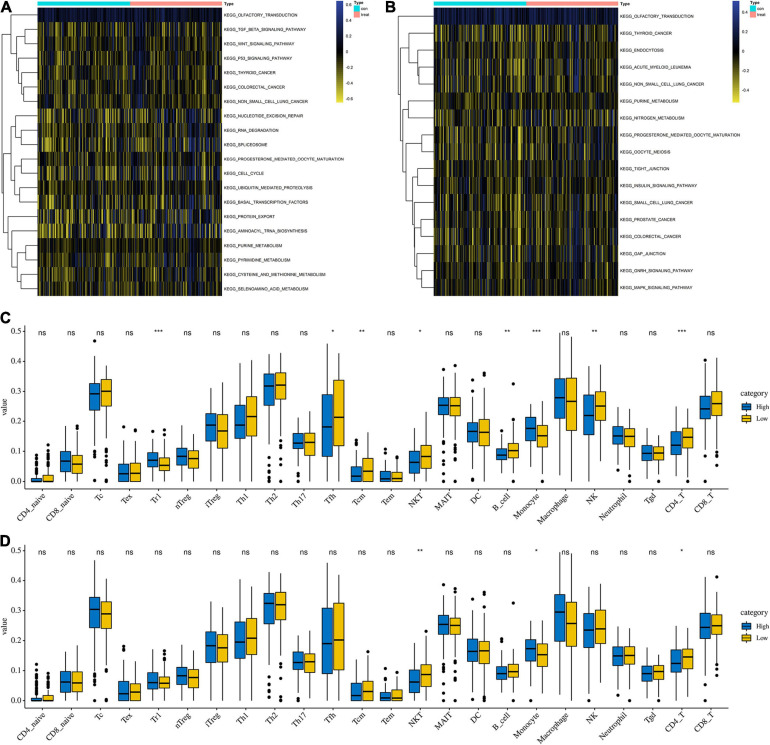
GSVA analysis and immune feature between low- and high-risk groups. **(A)** GSVA analysis between low- and high-risk groups based on overall survival signature. **(B)** GSVA analysis between low- and high-risk groups based on progress free-survival survival signature. **(C)** Comparison of immune cell infiltration between low- and high-risk groups based on overall survival signature. **(D)** Comparison of immune cell infiltration between low- and high-risk groups based on progress free-survival signature.

To explore the functions of differentially expressed parental genes in APA, GSVA analysis was conducted to predict the possible functions of APA events (*p* < 0.05). Two heatmaps were used to show differences between high- and low-risk groups (Figures 5A,B). The wnt signaling pathway, p53 signaling pathway, RNA degradation, and nucleotide excision repair were enriched in the high-risk group, which played a vital role in tumorigenesis. Meanwhile, we found that other malignant tumors were also enriched in the high-risk group, including colorectal cancer, prostate cancer, and small cell lung cancer, which suggested that the APA signature was positively related to the process of these kinds of tumors. Additionally, the difference in immune cell infiltration were also observed. Among 24 types of immune cells, the NKT cell, monocyte, and CD4 T cell were significantly different between low- and high-risk groups no matter if they were the in OS signature and PFS signature (Figures 5C,D). Moreover, the filtration of Tr1 cells, Tfh cells, Tcm cells, B cells, and NK cells was distinct between low OS group and high OS group (Figure 5C).

### Subgroup Analyses of Signatures in Different Subgroups

To study the ability of prognostic signatures in different clinical subgroups, the Kaplan-Meier survival curves in several subgroups were constructed (Supplementary Figures 3, 4). For the OS signature, in all 16 subgroups, survival analyses showed that patients in the low-risk group have a favorable OS than high-risk patients (Supplementary Figures 3A–H). Log-rank tests in all subgroups were statistically significant (all *p*-values < 0.05). Similarly, for the PFS signature, subgroup analyses indicated that low-risk patients had a better prognosis than high-risk patients (all *p*-values < 0.05) (Supplementary Figures 4A–H). Generally, subgroup analyses showed the stability and robustness of APA-based signatures, which further verified that APA events can serve as satisfactory prognostic predictors.

### Development of Two Clinical-APA Nomograms

**FIGURE 6:**
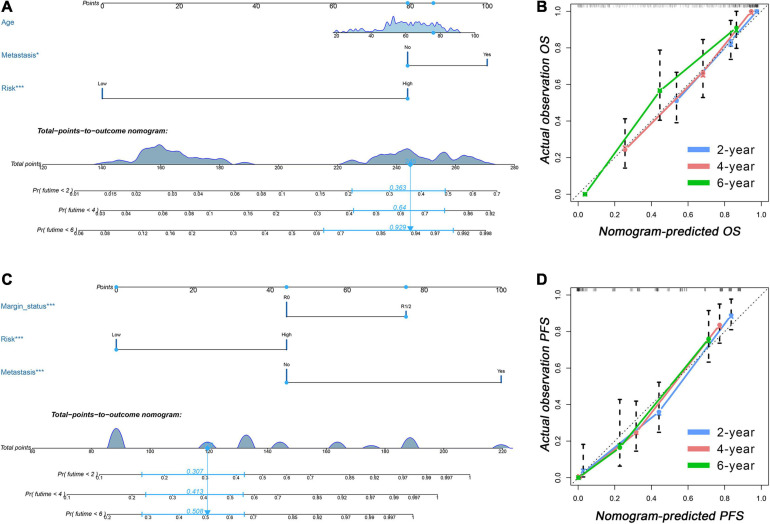
Clinical-APA nomograms for sarcoma. **(A)** A nomogram incorporated APA signature and prognostic clinical data was established to predict OS of sarcoma patients. **(B)** Calibration curves showed that nomogram-predicted OS were highly consistent with actual OS. **(C)** A nomogram incorporated APA signature and prognostic clinical data was established to predict PFS of sarcoma patients. **(D)** Calibration curves showed that nomogram-predicted PFS were highly consistent with actual PFS. APA, alternative polyadenylation; OS, overall survival; PFS, progress free-survival.

The results of the univariate Cox analysis indicated that APA signature, age, metastasis status, margin status, and multifocal indicator were OS-related variables (*p* < 0.05) (Table 1), while APA signature, metastasis status, margin status, and multifocal indicator were PFS-related variables (Table 2). We incorporated the above significant variables into the multivariate Cox analyses. The results indicated that both APA-based OS signature and APA-based PFS signature were robust prognostic predictors and were independent of clinical data (Tables 1, 2). Meanwhile, age and metastasis status were confirmed as independent OS-related variables (Table 1), while metastasis and margin status were independent PFS-related variables (Table 2). Two nomograms were developed to predict the OS and PFS, respectively (Figures 6A,C). In the nomogram, values for individual patients are located along the variable axes, and a line is drawn upward to the point axis to determine the number of points assigned for each variable. There is a total point line at the bottom of the nomogram, and each variable score is summed to give the total points. Then, a vertical line is drawn from the total point scale to the bottom three lines to obtain the prognosis at 2-, 4-, and 6-years. For example, in the nomogram of PFS, a patient with low risk, no distant metastasis, and a margin status of R1/2 would have a total score of 120, and his 2-year, 4-year, and 6-year PFS rates would be 30.7, 41.3, and 50.8%, respectively. The C-index for OS nomogram was 0.813 and 0.809 for the PFS nomogram, which means that both nomograms have favorable discrimination. The calibration curves for 2-, 4-, and 6-years indicated that the nomogram-predicted outcomes were highly consistent with actual observation outcomes, no matter whether in OS or PFS nomogram (Figures 6B,D).

**TABLE 1:** Overall survival analysis of APA signature and clinical data for sarcoma.

	Univariate Cox analysis			Multivariate Cox analysis		
		
	HR	95% CI	*P*-value	HR	95% CI	*P*-value
Risk (Low)	0.093	0.054–0.159	0.000	0.091	0.038–0.217	0.000
Age	1.020	1.005–1.036	0.010	1.025	1.002–1.049	0.032
Sex (Male)	0.855	0.572–1.277	0.443			
Race						
Asian						
Black	1.085	0.132–8.924	0.939			
White	0.791	0.108–5.773	0.817			
Histological						
DLP						
LMS	0.842	0.512–1.386	0.500			
MFS	0.703	0.328–1.507	0.364			
Other	0.739	0.319–1.710	0.479			
UPS	0.901	0.481–1.691	0.746			
Tumor site (Other)	1.229	0.792–1.906	0.357			
Metastasis (Yes)	3.014	1.834–4.954	0.000	2.410	1.335–4.349	0.004
Margin status (R1/2)	2.554	1.668–3.910	0.000	1.416	0.786–2.550	0.247
Multifocal indicator (Yes)	2.404	1.502–3.847	0.000	1.085	0.517–2.278	0.830
Radiotherapy (Yes)	0.988	0.619–1.579	0.961			
Pharmacotherapy (Yes)	1.382	0.815–2.341	0.230			

*APA, alternative polyadenylation; HR, hazard ratio; CI, confidence interval; DLP, dedifferentiated liposarcoma; LMS, leiomyosarcoma; MFS, myxofibrosarcoma; UPS, undifferentiated pleomorphic sarcoma.*

**TABLE 2:** Progress free-survival analysis of APA signature and clinical data for sarcoma.

	Univariate Cox analysis			Multivariate Cox analysis		
		
	HR	95% CI	*P*-value	HR	95% CI	*P*-value
Risk (Low)	0.251	0.178−0.356	0.000	0.221	0.138−0.354	0.000
Age	1.006	0.995−1.018	0.267			
Sex (Male)	1.096	0.797−1.506	0.573			
Race						
Asian						
Black	3.146	0.402−24.609	0.275			
White	2.954	0.412−21.170	0.281			
Histological						
DLP						
LMS	0.926	0.619−1.386	0.708			
MFS	0.730	0.395−1.349	0.315			
Other	0.807	0.421−1.547	0.519			
UPS	0.870	0.528−1.433	0.584			
Tumor site (Other)	0.990	0.706−1.388	0.952			
Metastasis (Yes)	6.001	3.948−9.122	0.000	6.412	4.012−10.247	0.000
Margin status (R1/2)	2.202	1.565−3.099	0.000	2.589	1.634−4.100	0.000
Multifocal indicator (Yes)	1.972	1.307−2.975	0.001	1.567	0.850−2.887	0.150
Radiotherapy (Yes)	1.108	0.769−1.597	0.581			
Pharmacotherapy (Yes)	1.437	0.929−2.222	0.103			

*APA, alternative polyadenylation; HR, hazard ratio; CI, confidence interval; DLP, dedifferentiated liposarcoma; LMS, leiomyosarcoma; MFS, myxofibrosarcoma; UPS, undifferentiated pleomorphic sarcoma.*

### A Network of Prognostic APA Events and APA Factors

**FIGURE 7:**
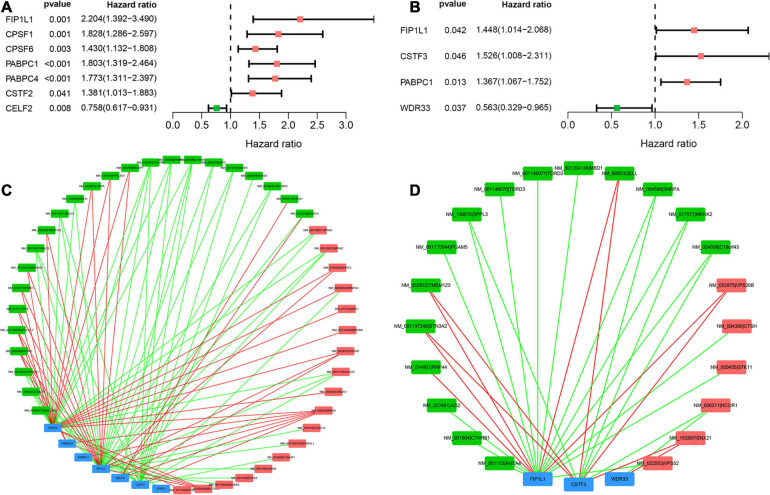
Survival-associated APA factors and APA events correlation network in sarcoma. **(A)** Seven OS-related APA factors identified by univariate Cox analysis. **(B)** Four PFS-related APA factors identified by univariate Cox analysis. **(C)** Regulatory network of OS-related APA factors and APA events. Blue rectangles means APA factors, green rectangles means protective APA events, and red rectangles means risk APA events. Green line means negative correlation between the PDUI of APA events and the expression of APA factors. **(D)** Regulatory network of PFS-related APA factors and APA events. Blue rectangles means APA factors, green rectangles means protective APA events, and red rectangles means risk APA events. Green line means negative correlation between the PDUI of APA events and the expression of APA factors. APA, alternative polyadenylation; OS, overall survival; PFS, progress free-survival.

According to the univariate Cox analysis, 61 and 38 APA events were OS- and PFS-related biomarkers, respectively. In addition, the expression of 28 core APA factors was extracted. In total, seven APA factors were identified as OS-related APA factors, and four APA factors were identified as PFS-related APA factors (Figures 7A,B). The correlation between APA events and APA factors was studied. Finally, two APA-APA factors regulatory networks were established (Figures 7C,D). The regulatory between APA events and APA factors were initially elucidated. The first network contained seven APA factors and 42 APA events to show the regulatory mechanism of OS-related APA events (Figure 7C). Another network including three APA factors and 21 APA events was established to show the regulatory mechanism of PFS-related APA events (Figure 7D). Throughout the two networks, we can find that one APA factor can regulatory more than one APA event. Additionally, one APA event can be regulated by more than one APA factor even two opposite regulations.

### Pan-Cancer Analyses of FIP1L1-VPS26B Regulating Relationship

**FIGURE 8:**
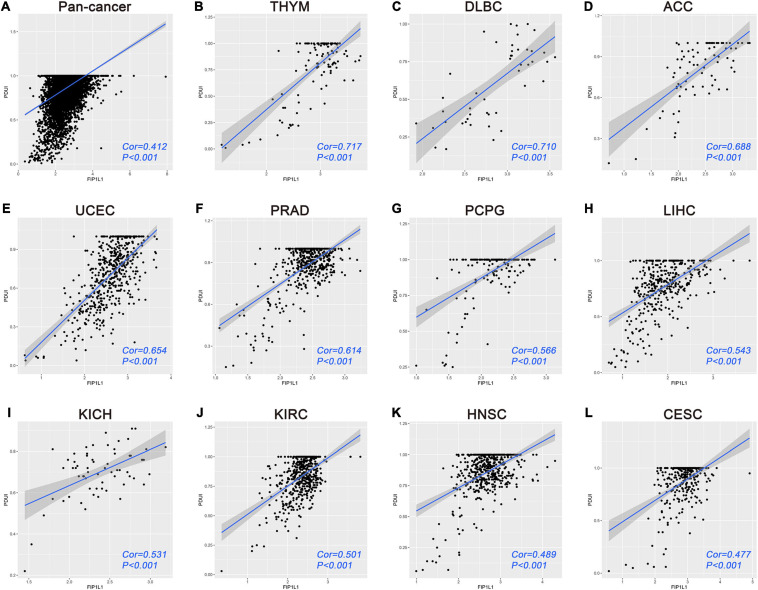
Pan-cancer analyses of FIP1L1-VPS26B regulating relationship. **(A)** Correlation analysis of FIP1L1-VPS26B in 9087 patients indicated that the expression of FIP1L1 is significantly associated with the PDUI of VPS26B. **(B–L)** Correlation results of top 11 cancer in 30 cancer types.

In two regulatory networks, one interesting regulating relationship presented itself for our attention. According to the survival analyses, APA events of VPS26B were identified as overlapping independent prognostic APA events between OS and PFS. In addition, FIP1L1 was shown to be significantly associated with both OS and PFS. More importantly, in the regulatory network, the expression of FIP1L1 was significantly related to the PDUI of VPS26B. Hence, we speculated this regulating relationship may have an important role in malignancy to comprehensively understand this regulating relationship in malignancy. We performed a pan-cancer analysis based on the UCSC Xena browser and TC3A database. A total of 9087 patients were included, including 30 cancer types. The Pearson correlation analysis indicated that the expression of FIP1L1 is significantly associated with the PDUI of VPS26B (Figure 8A). Furthermore, we analyzed the correlation of FIP1L1-VPS26B in specific cancers. Among 29 types (sarcoma was excluded), only four tumor cohorts showed that there was no significant correlation between FIP1L1 and VPS26B. The correlation coefficient is the highest in the TCGA-THYM cohort (cor = 0.717), followed by TCGA-DLBC (cor = 0.710) (Figures 8B–L).

## Discussion

Alternative polyadenylation is a RNA-processing mechanism that generates distinct 3’ termini on mRNAs and other RNA polymerase II transcripts (Tian and Manley, 2017). It is widespread across all eukaryotic species and is recognized as a major mechanism of gene regulation (Tian and Manley, 2017). The role of APA in human cancers is only beginning to be appreciated, which shows the potentially robust predictive value for tumor patients. More importantly, compared with genomic data only, APA signature may have a better predictive ability in patients’ prognosis (Xia et al., 2014; Feng et al., 2018).

In the present study, we mainly focused on the profiling of the prognostic value of APA events to explore the utilization of APA signatures in predicting the outcome of sarcoma patients. In total, 259 primary sarcoma patients from TC3A database and TCGA dataportal were included. A total of 61 and 38 APA events were determined as OS- and PFS-related biomarkers, respectively. Two 16 APA-based signatures were built and showed favorable prognostic predictors, with all AUC values above 0.900 for OS signature and 0.820 for the PFS signature. Combined with independent prognostic clinical variables, two APA-clinical nomograms were developed and showed satisfactory discrimination and calibration. Finally, combined survival and correlation network analyses between APA events and APA factors, our research uncovered the underlying mechanism of APA events involved in patient prognosis. To our knowledge, the present study is the first study to establish APA signatures for predicting the survival of sarcoma patients.

Identifying effective biomarker and constructing an ideal prognostic signature or nomogram has long been the focus of oncologists, which can individually predict the specific outcomes to guide the management of tumor patients. At present, a great number of biomarkers have been reported, and several prognostic models for sarcoma were constructed (Benassi et al., 2015; Callegaro et al., 2017, 2019; Yang et al., 2017; Huang et al., 2019; Raut et al., 2019; Zhang et al., 2019a; Gu et al., 2020; Hu et al., 2020; Zhu et al., 2020). For example, MS Benassi et al. (2015) reported that the expression of IGFBP7, considered a tumor stroma marker in mesenchymal-derived cells, was highly prognostic in poor metastasis-free survival for soft tissue sarcoma. Additionally, clinicopathological variables, ncRNA data, or immune cells were also confirmed as predictors and used to develop prognostic models (Callegaro et al., 2017, 2019; Yang et al., 2017; Huang et al., 2019; Raut et al., 2019; Zhang et al., 2019a; Gu et al., 2020; Hu et al., 2020; Zhu et al., 2020). Nevertheless, clinical practice is not optimistic. Current research mainly focused on the prognostic role of clinical data or gene level, overlooking the transcriptional level. As one of the important post-transcriptional regulatory mechanisms, APA events have shown a potentially robust predictive value for tumor patients (Venkat et al., 2020; Zhang et al., 2020b). Our study confirmed that APA events are valuable predictors for sarcoma patients, no matter whether in general patients or several subgroups. In the mechanism, APA play a vital role in gene regulation and diverse cellular processes, including mRNA metabolism, protein diversity, and protein localization (Tian and Manley, 2017).

In total, 31 APA events, including 15 APA events for OS signature only, 15 APA events for PFS signature only, and 1 APA event for both OS and PFS signatures, were used to construct two signatures. Among the corresponding genes of these APA events, most of which were associated with tumorigenesis and progression. For example, by activating the EMT and non-canonical WNT signaling, ABI1 can drive the tumorigenesis of prostate cancer (Nath et al., 2019). Additionally, dysregulation of ABI1 was confirmed associated with the prognosis of gastric cancer, epithelial ovarian cancer, and breast cancer (Cui et al., 2010; Wang et al., 2011; Zhang et al., 2015). Another widely studied gene is MCM2, which is a vital initiation factor for DNA replication in humans. It also presents in the nucleus and is overexpressed in proliferating cells. The prognostic value of MCM2 was confirmed in pancreatic cancer, lung cancer, multiple myeloma, and oral cancer (Ramnath et al., 2001; Torres-Rendon et al., 2009; Deng et al., 2020; Quan et al., 2020). Significantly, the APA event of VPS26B was determined as an overlapping independent prognostic biomarker for sarcoma patients. Nevertheless, few studies reported the role of VPS26B or related regulation in cancers. Future studies may focus on its prognostic value in other tumors and their regulatory role in tumors.

In our research, APA and APA factor regulatory networks were also constructed. Various of regulating relationships between them were identified. Interestingly, one core regulating relationship was confirmed, and the same trend was also confirmed in the pan-cancer analysis. As a novel regulator for APA events, FIP1L1 can regulate the 3’UTR lengthening of leukemia-associated genes, including NRAS, BAALC, and MAPKAPK3 (Davis et al., 2018). Interestingly, both overexpression and knockdown of FIP1L1 are harmful to leukemia cells, demonstrating that mild alteration of gene expression may dramatically impact on cell fitness (Davis et al., 2018). In addition to FIP1L1, eight APA factors were included incorporated into regulatory networks. The mechanism of part of factors has been preliminarily elucidated. As a vital cleavage/polyadenylation factor, CSTF2 can shorten the length of 3’UTR RAC1 in human urothelial carcinoma of the bladder by mediating slow transcriptional elongation at RAC1 (Chen et al., 2018). Moreover, F6 was considered as a vulnerability target for breast cancer patients (Binothman et al., 2017). Despite this research, the regulatory mechanism between APA and APA factors in sarcoma remains unclear. More than 100 potential regulating relationships were detected in our research, which pointed out the directions for future research.

Although the strict bioinformatic and statistical methods were used and the prognostic value of APA events in sarcoma patients have been discovered in our research, there are some limitations. First, due to the relative rarity of sarcoma, only bioinformatic analyses were used in this study, and no further experimental analysis based on clinical samples was performed to validate our results. Second, external validation is vital for clinical application of prognostic signatures or nomograms. Unfortunately, no available independent cohort can be obtained from other databases. Thirdly, no available normal sample can be incorporated into our study. Therefore, the diagnostic value and the potential carcinogenic effect of APA events cannot be studied in this research. Finally, the data used in the study were obtained from public datasets from which the available clinical data is limited and incomplete. Therefore, some prognostic variables, such as tumor grade and size, were not available and were not analyzed in the present study.

## Conclusion

Our data revealed the prognostic value of survival−related APA events for sarcoma. Some key APA factors might play essential roles in tumor initiation and progression by regulating the corresponding APA events. Our findings might offer a new prospect for effective therapies targeted at APA events for sarcoma.

## Data Availability Statement

Publicly available datasets were analyzed in this study. This data can be found here: https://xenabrowser.net/ and tc3a.org.

## Author Contributions

CH, YX, and CL performed the data analysis and wrote the manuscript. JL, CL, JD, and XT contributed to the data analysis and manuscript revision. CH, TY, BC, and YD contributed to literature search and data extraction. CH and YX conceived and designed the study. All authors have read and approved the final version of the manuscript.

## Conflict of Interest

The authors declare that the research was conducted in the absence of any commercial or financial relationships that could be construed as a potential conflict of interest.

## Footnotes

1 https://xenabrowser.net/

2 http://metascape.org

## Supplementary Material

Supplementary Figure 1 LASSO analysis of OS- and PFS-related APA events. **(A,C)** Dotted vertical lines were drawn at the optimal values by using the minimum criteria. **(B,D)** LASSO coefficient profiles of the candidate OS **(B)** and PFS **(D)-**related alternative polyadenylation events. A coefficient profile plot was produced against the log λ sequence. LASSO, least absolute shrinkage and selection operator; APA, alternative polyadenylation; OS, overall survival; PFS, progress free-survival.

Supplementary Figure 2 The distribution of clinical covariates across low- and high-risk groups. **(A)** Overall survival signature; **(B)** Progress free-survival signature. DLP, dedifferentiated liposarcoma; LMS, leiomyosarcoma; MFS, myxofibrosarcoma; UPS, undifferentiated pleomorphic sarcoma.

Supplementary Figure 3 Subgroup analyses of OS signature. Survival curves showed that high-risk patients were significant worse OS than low-risk patients in subgroups of age **(A)**, sex **(B)**, cancer histological type **(C,D)**, metastatic status **(E)**, tumor site **(F)**, margin status **(G)**, and multifocal indicators **(H)**. OS, overall survival.

Supplementary Figure 4 Subgroup analyses of PFS signature. Survival curves showed that high-risk patients were significant worse PFS than low-risk patients in subgroups of age **(A)**, sex **(B)**, cancer histological type **(C,D)**, metastatic status **(E)**, tumor site **(F)**, margin status **(G)**, and multifocal indicators **(H)**. PFS, progress-free survival.
