# Supplementary figures and images for: Construction of Two Alternative Polyadenylation Signatures to Predict the Prognosis of Sarcoma Patients
Source: Front Cell Dev Biol. 2021 Jun 14;9:595331. doi: 10.3389/fcell.2021.595331 (PMC8236624; doi:10.3389/fcell.2021.595331)

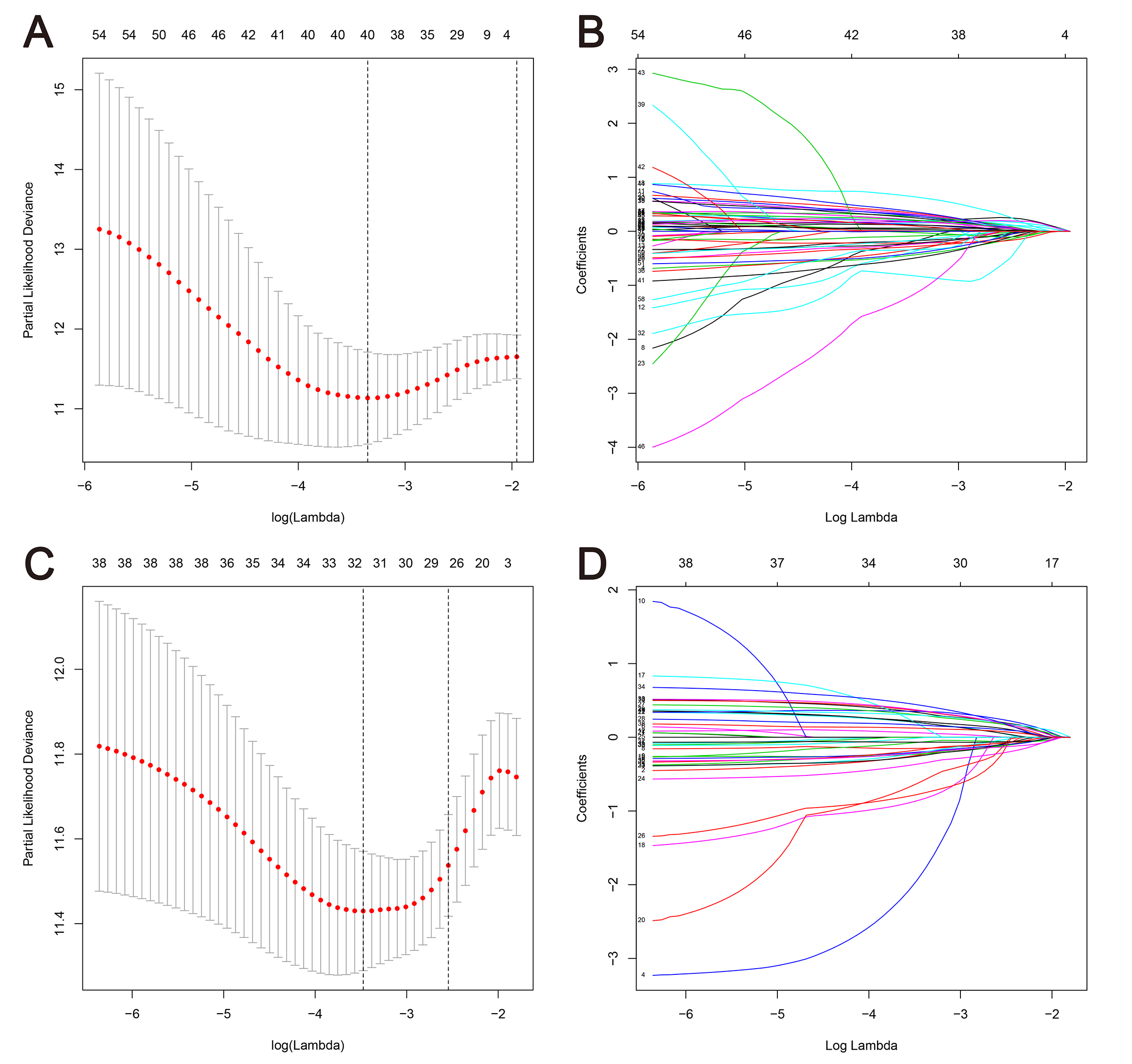

Supplement: Supplementary Figure 1 — LASSO analysis of OS- and PFS-related APA events. (A,C) Dotted vertical lines were drawn at the optimal values by using the minimum criteria. (B,D) LASSO coefficient profiles of the candidate OS (B) and PFS (D)-related alternative polyadenylation events. A coefficient profile plot was produced against the log λ sequence. LASSO, least absolute shrinkage and selection operator; APA, alternative polyadenylation; OS, overall survival; PFS, progress free-survival. [file Image_1.TIF]

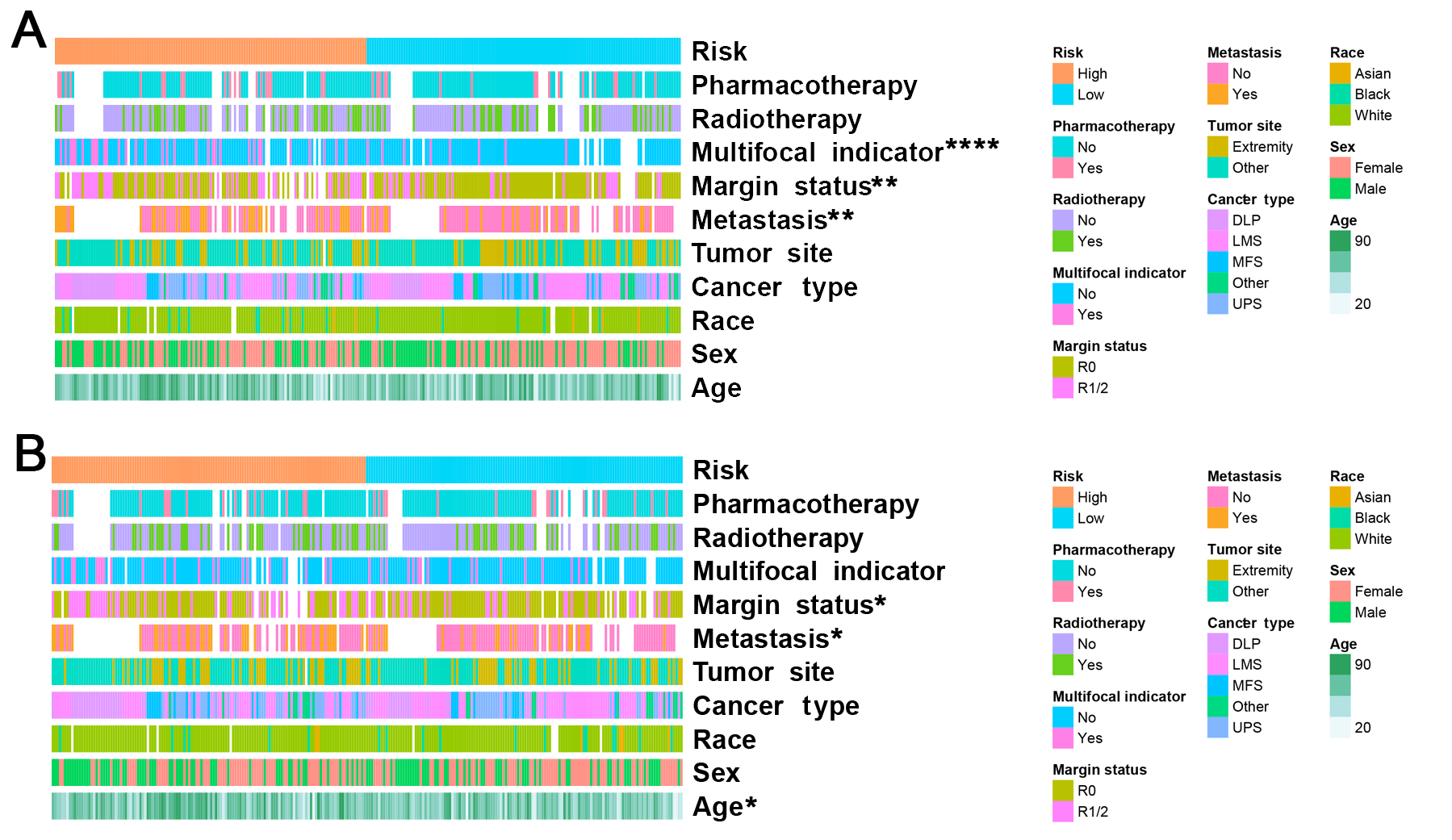

Supplement: Supplementary Figure 2 — The distribution of clinical covariates across low- and high-risk groups. (A) Overall survival signature; (B) Progress free-survival signature. DLP, dedifferentiated liposarcoma; LMS, leiomyosarcoma; MFS, myxofibrosarcoma; UPS, undifferentiated pleomorphic sarcoma. [file Image_2.TIF]

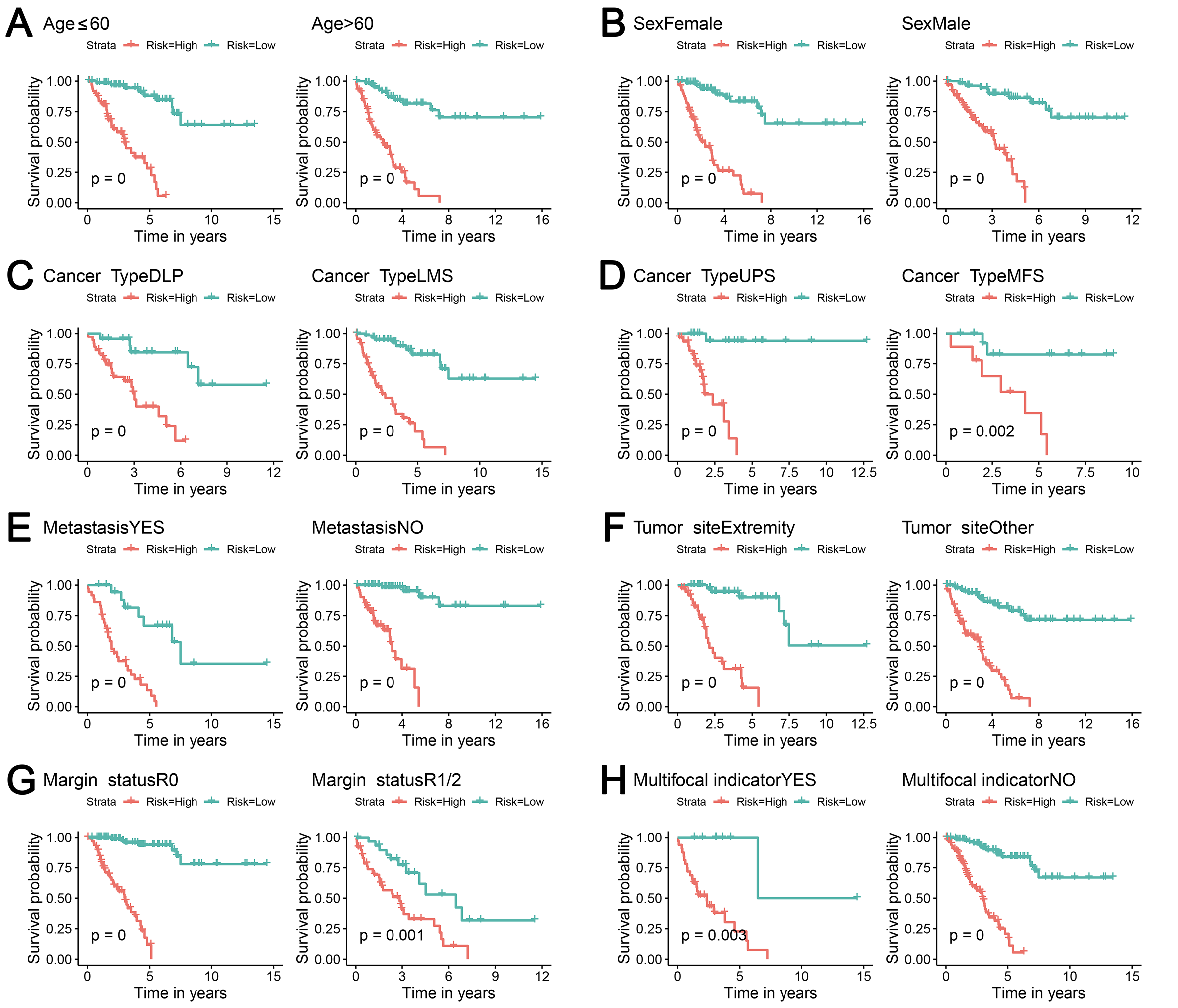

Supplement: Supplementary Figure 3 — Subgroup analyses of OS signature. Survival curves showed that high-risk patients were significant worse OS than low-risk patients in subgroups of age (A), sex (B), cancer histological type (C,D), metastatic status (E), tumor site (F), margin status (G), and multifocal indicators (H). OS, overall survival. [file Image_3.TIF]

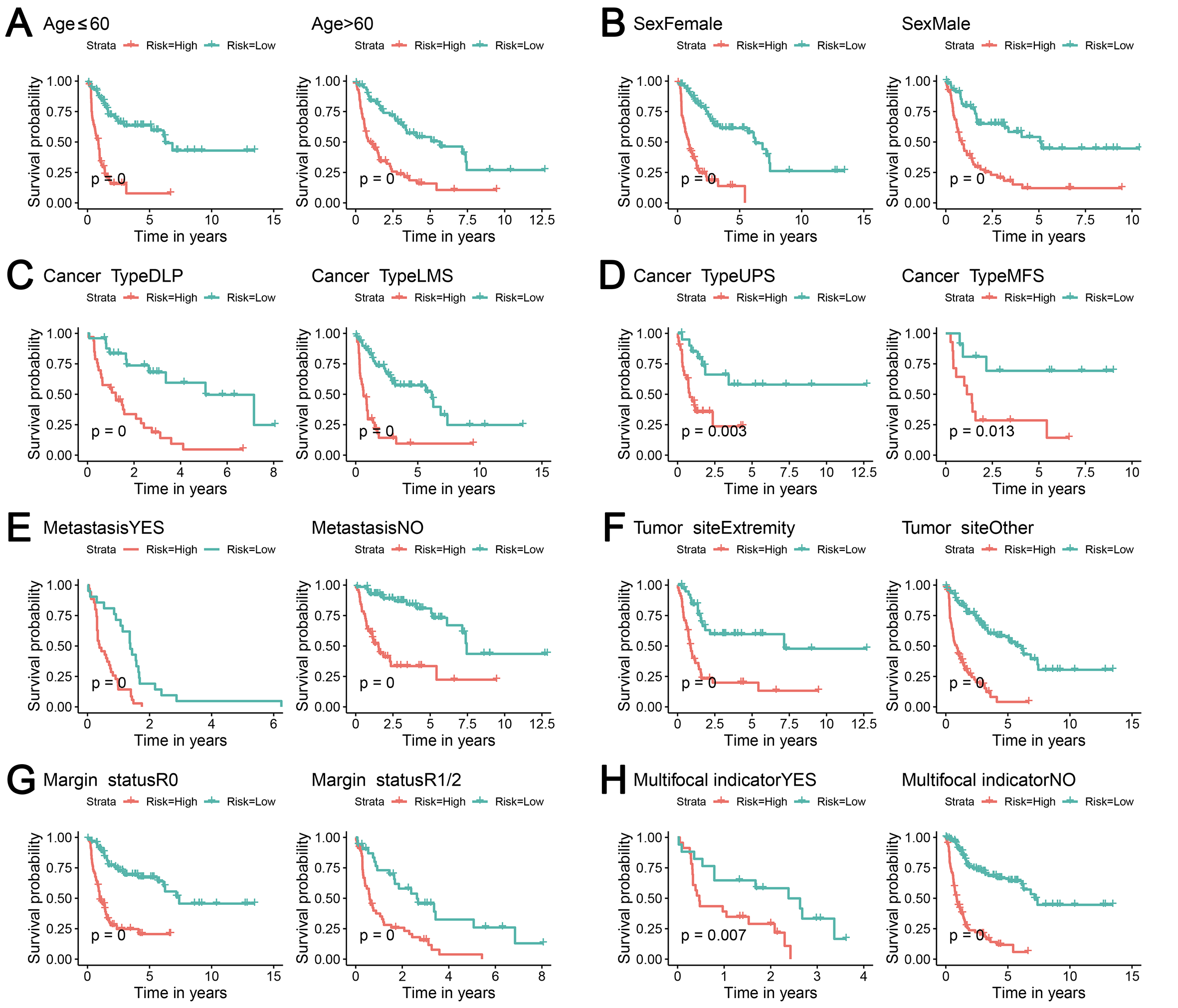

Supplement: Supplementary Figure 4 — Subgroup analyses of PFS signature. Survival curves showed that high-risk patients were significant worse PFS than low-risk patients in subgroups of age (A), sex (B), cancer histological type (C,D), metastatic status (E), tumor site (F), margin status (G), and multifocal indicators (H). PFS, progress-free survival. [file Image_4.TIF]
